# Pediatric Heterotopic Ossification: A Comprehensive Review

**DOI:** 10.1007/s12178-023-09862-y

**Published:** 2023-08-17

**Authors:** Alexander R. Markes, Nikit Venishetty, Andrew Gatto, Ishaan Swarup

**Affiliations:** 1 Department of Orthopaedic Surgery, University of California-San Francisco, 1500 Owens Street, San Francisco, CA USA; 2 Texas Tech University Health Sciences Center El Paso, El Paso, TX USA; 3 Touro University California, Vallejo, CA USA

**Keywords:** Pediatric heterotopic ossification, Pediatric heterotopic ossification prophylaxis, Pediatric heterotopic ossification management, Pediatric heterotopic ossification risk factors

## Abstract

**Purpose of Review:**

The purpose of this review is to provide a comprehensive analysis of heterotopic ossification (HO) in pediatric patients, including an in-depth examination of the risk factors associated with this condition, current prophylactic measures, and available management strategies.

**Recent Findings:**

HO is a medical disorder in which bone tissue inexplicably develops in soft tissues such as muscles and tendons. It involves the formation of mature, lamellar bone in extra-skeletal soft tissue, and its formation is influenced by oxygen tension, pH, the availability of micronutrients, and mechanical stimulation. HO has many cellular origins, with the most common theory being multipotent cells in local tissue. The diagnosis of HO is typically made based on exam, radiographs, and CT. Management includes both prophylactic nonsurgical options and surgical resection for severe or recalcitrant cases.

**Summary:**

The review highlights the incidence, risk factors, and management strategies associated with HO in pediatric patients. HO is a rare condition in children, with severe neurologic injury being the most common cause. Pediatric patients most commonly develop HO following severe neurologic injury, followed by trauma and surgery. Current prophylactic measures, include nonsteroidal anti-inflammatory drugs and radiation therapy though limited literature on their use in the pediatric population exists. For recalcitrant symptomatic cases, wide surgical resection can be considered but has a higher risk profile and associated morbidity. This review highlights the need for further pediatric specific research to inform guidelines and management strategies for this debilitating condition.

## Introduction

Heterotopic ossification (HO) is a medical disorder in which bone tissue develops in soft tissues like muscles and tendons [1••]. HO involves the formation of mature, lamellar bone in extra-skeletal soft tissue [1••, 2, 3, 4••, 5, 6]. The most frequent causes of heterotopic ossification include joint replacement, heat injury, fractures of the elbow and acetabulum, spinal cord injury, traumatic brain injury, and blast trauma [5, 7]. While some HO lesions may be minor and clinically insignificant, others may cause significant morbidity [4••]. This review aims to provide a comprehensive analysis of the incidence of HO in pediatric patients, as well as an in-depth examination of the risk factors associated with this condition, current prophylactic measures, and available management strategies.

## Epidemiology

Heterotopic ossification (HO) exhibits a lower incidence in children compared to adults, with reports ranging between 4 and 22% [5, 8, 9•, 10]. In a comprehensive single institutional study of 643 patients admitted for neuropediatric rehabilitation, HO was diagnosed in 5% (*n* = 32) of patients, with the hip joint most frequently involved (*n* = 17) followed by the shoulder (*n* = 9), elbow (*n* = 9), and knee (*n* = 6) [5]. Rarely, a patient will develop multiple sites of HO [5, 11, 12]. Unlike in adults, there is no male predominance in pediatric HO, though increased age may be associated with higher likelihood [5, 9•, 10, 13, 14]. Pediatric patients most commonly develop HO following severe neurologic injury, followed by trauma and surgery [5].

## Risk Factors

Heterotopic ossification can be a disabling complication for severe central nervous system insults, including traumatic and anoxic brain injury, encephalitis, and spinal cord injury [5, 10, 15]. Kluger et al. reported the development of HO in 7.9% of severe cases of traumatic brain injury and 3.8% of near-drowning cases [5]. Preexisting neurologic conditions, such as cerebral palsy, may predispose patients to HO development depending on the degree of impairment [13, 16].

Traumatic burn injury is a well-documented cause of HO with incidence as high as 35% when patients are screened, although only 0.25% cause significant impairment [11, 17]. The risk is tightly associated with total burn surface area with greatest prevalence in the elbow [11, 12, 17]. Besides burns on TBI, other traumatic events such as fractures, dislocations, and ligament sprains can also precede HO [3, 18–21].

Surgery has also been seen as a risk factor for development of HO in pediatrics and adults [22]. HO has been reported in pediatric internal fracture fixation and elbow arthroscopy [9•, 23–27]. Feroe et al. reported HO in 4.5% of pediatric patients within 2 years of hip arthroscopy, most often pericapsular [9•, 28]. Elbow arthroscopy appears to be a less common cause of HO, although this may change as procedures become more complex [24, 25]. The use of mechanical hardware has also been implicated in the development of HO, particularly in rigid intramedullary fixation for diaphyseal femoral shaft fractures with a protruding proximal rod [29–31]. Sutphen et al. found HO in 24% of patients with rigid femoral nails versus 5.9% with submuscular plate and none with flexible nail [27]. While adults have reported HO related to traction pins, no such cases have been reported in pediatric use [26]. Though very uncommonly performed in the pediatric population, total joint arthroplasty is a common cause of HO in adults that has additionally been well studied and documented [4••].

In extremely rare cases, HO can be attributed to a genetic cause. Fibrodysplasia ossificans progressiva (FOP), for instance, is a condition with a prevalence of 1.36 per million where polytopic HO is typically preceded by other congenital malformations [32–34]. Progressive osseous heteroplasia bears resemblance to FOP but emerges in infancy and is characterized by dermal ossifications that progress into deeper tissue [6]. Additionally, symptomatic HO has been associated with Albright hereditary osteodystrophy and ankylosing spondylitis [35, 36].

## Clinical Presentation and Imaging

**Fig. 1:**
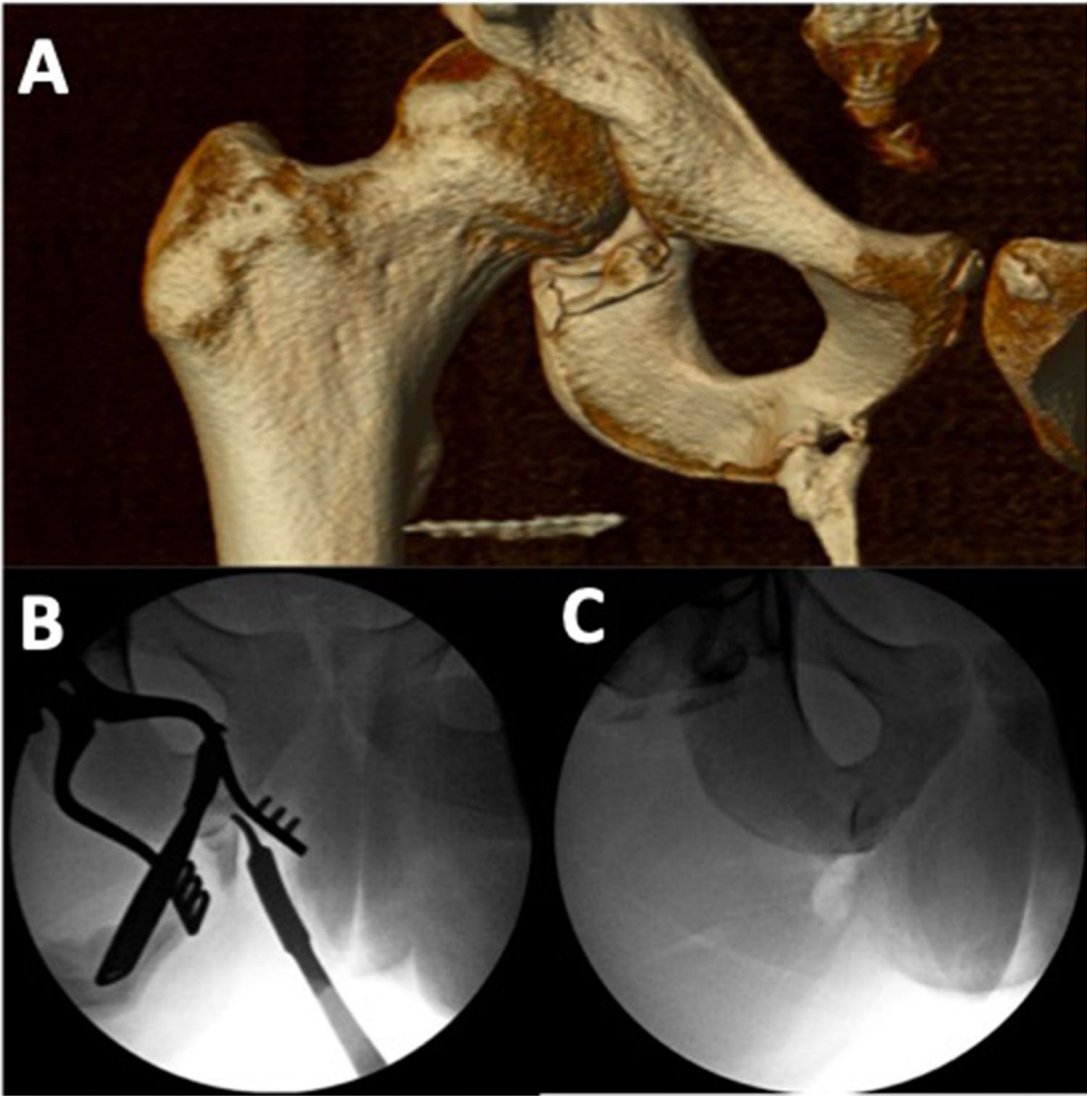
**A** Computed tomography 3D reconstructions and **B** intra-operative fluoroscopy demonstrating heterotopic ossification of the right femur and ischium in a 16-year male who suffered severe traumatic brain injury from a motor vehicle accident. **C** Intra-operative fluoroscopy demonstrating resection of the prior ischial heterotopic ossification

The onset of HO is variable, presenting within 3 weeks to 20 months after an inciting injury, with typical presentation within 4 months [5, 11, 12, 18, 19]. Though HO progresses more rapidly in children, pediatric cases are more likely to follow an indolent course [11, 12]. Severe HO predominantly manifests with sharp pain, decreased range of motion, and localized soft tissue swelling. Rare cases of bilateral joint involvement have been reported in neurologic and burn injury [12, 15, 37]. Clinical presentation can be further influenced by the location and size of the lesion, with findings including fever, palpable mass, neurovascular compression, and gait abnormalities [3, 11, 12, 15, 37] (Fig. 1).

Radiography is the primary modality for diagnosing HO, demonstrated as a peripheral zone of ossification which matures to a well-defined cancellous bone [38••]. HO typically only affects soft tissue, but it can also adhere to the surface of the bone (also termed parosteal HO) [4••]. To its disadvantage, radiography cannot detect HO until 4 to 8 weeks after symptom onset [12, 39]. For early diagnosis, triphasic nuclear bone scans and ultrasound are more sensitive [5, 17, 39]. Ultrasound is particularly advantageous due to bedside application, lack of radiation, and ability to quantitatively monitor progression through gray-scale variability [40]. Serum alkaline phosphatase can be elevated in the setting of HO, though was only seen increased above age-related normal levels in 16% (4/24) of patients by Kluger et al [5]. In magnetic resonance imaging (MRI) studies, the acute phase of HO has increased tissue vascularization and density [38••]. When HO is mature, it manifests as hyperintense cancellous fat that is bordered by hypointense cortical bone on T1 and T2 weighted imaging [41]. Computed tomography provides the most precise localization of HO and its relationship to surrounding tissues and can be useful for surgical planning [5, 12, 17, 42].

## Histology

While the exact etiology of HO formation is unclear, studies suggest that HO results from the transformation of progenitor cells into osteogenic precursor cells as a result of cell-mediated interactions with the local tissue environment, which is influenced by oxygen tension, pH, the availability of micronutrients, and mechanical stimulation [7, 42–44]. Tissues of HO are usually disorganized and inhomogeneous [43]. HO has been thought to have many cellular origins including satellite cells, smooth muscle cells, and even endothelial cells, with the most common theory it being multipotent cells in local tissue [38••, 45–47]. An osteogenic precursor, an inducing agent, and an environment that is favorable for osteogenesis are all prerequisites for the creation of HO, which will eventually lead to proliferation and the formation of bone [38••, 41, 43].

According to histological investigations, both the endochondral process, which needs a cartilage template, and the intramembranous process, which does not necessitate cartilage production, can result in heterotopic ossification [48]. HO is defined by zonal distribution of proliferating fibroblasts that are surrounded by metaplastic trabecular bone [4••, 43, 49]. Early lesions have a high mitotic index with plump fibroblasts [44, 50]. Lesions can be several centimeters in size, and can comprise of exuberant, cellular granulation tissue that can mimic a sarcoma [7]. The presence of osteoblasts distinguish HO from dystrophic calcification [43].Within 6 weeks, these lesions develop a characteristic zonal pattern with the outer portion of the mass populated with dense lamellar bone arranged as a pseudocortex with spicules of bone are progressively thinner toward the center of the lesion. After 6 months to a year, the lesions develop into an orderly arrangement of thick, mature trabecular bone [50].

## Prophylaxis

### Nonsteroidal Anti-Inflammatory Drugs

Though the indications, role, and efficacy of pharmacologic therapy for HO prophylaxis is debated, the primary treatment modality is nonsteroidal anti-inflammatory drugs (NSAIDs) [5, 8]. NSAIDs are inhibitors of COX-2 which can inhibit HO formation through two proposed mechanisms. (1) Inhibition of COX-2 prevents it facilitating the differentiation of mesenchymal cells into osteoblasts and (2) COX inhibition can prevent prostaglandin formation which plays a role in angiogenesis needed for endochondral ossification during bone formation [51]. In adults and adolescents older than 15 years, the historical gold standard is indomethacin 25 mg three times a day for 6 weeks after surgery. This was popularized by Ritter et al., who demonstrated significant decrease in the incidence of HO in “high-risk” total hip arthroplasty candidates after institutional policy to treat all “high-risk” patients prophylactically with indomethacin [52]. No data regarding dosing of indomethacin in pediatric patients for HO exists; however, for children between the ages of 2 and 15, indomethacin has been tolerated in patients with inflammatory arthropathies at 1 to 2 mg/kg/day in 3 to 4 divided doses with maximum daily dose of 4 mg/kg/day or 200 mg/day, whichever is less [53].

Currently, there are no consensus guidelines for use of NSAIDs for prophylaxis against HO in the pediatric population. In general, most providers will consider pharmacologic prophylactic therapy in pediatric patients at high-risk as outlined above such as those who recently suffered traumatic brain injury admitted to neuropediatric rehabilitation unit and those who have suffered traumatic burn injuries involving an at-risk joint. In Kluger et al., they remark on a shift in institutional policy between 1995 and 1998 to begin using salicylates for patients in coma or persistent vegetative state with warm and painful swelling of a joint [5]. Though they are unable to offer conclusive proof of its efficacy, they comment that in the 231 patients they admitted for neuropediatric rehabilitation over this period, they observed no new cases of heterotopic ossification compared with 32 patients out of 643 admitted between 1985 and 1994 [5].

### Radiation

Low-dose radiation has been well studied in the adult literature as a prophylactic modality in the context of hip arthroplasty for primary HO occurrence or HO recurrence following resection [4••, 54]. For high-risk patients (those with hypertrophic osteoarthritis, ankylosing spondylitis, diffuse idiopathic skeletal hyperostosis, or prior HO), a single fraction of 700 cGy can be given either 24 h preoperatively or up to 72 h postoperatively with doses given outside this temporal window demonstrating a greater rate of HO [4••, 55, 56]. Radiation similarly to NSAIDs is thought to prevent osteoblast differentiation of mesenchymal pluripotent stem cells [57]. Though proven efficacious in limiting development of HO in the adult population in various settings such as post-op acetabular fracture fixation, it has a higher risk profile than indomethacin including arthrofibrosis, oncogenesis, and nonunion in the setting of fracture [4••, 58, 59]. Given these associated risks and the lack of data regarding safety and efficacy in the pediatric population, it is our understanding that the radiation is not routinely used for HO prophylaxis (Table 1).

**Table 1:** Considerations for prophylaxis of pediatric heterotopic ossification in high-risk* patients

Intervention	Dosing	Advantages	Limitations
Indomethacin	1 to 2 mg/kg/day in 3 to 4 divided doses with maximum daily dose of 4 mg/kg/day or 200 mg/day, whichever is less	Ease of administration, proven efficacy in adult populations	Limited data within pediatric populations, relative contraindications include presence of kidney disease, blood coagulation deficiencies, and dyscrasias
Salicylates	60 mg/kg of body weight, divided to maintain a mid-dose serum drug level of 15 to 20 mg/mL	Ease of administration, documented use in pediatric populations	Limited high-level data regarding efficacy in pediatric populations Relative contraindications include presence of kidney disease, blood coagulation deficiencies, and dyscrasias
Radiation	A single fraction of 700 cGy can be given either 24 h preoperatively or up to 72 h	Proven efficacy in adult populations	Not routinely used in pediatric populations High-risk profile including arthrofibrosis, oncogenesis, and nonunion in the setting of fracture

^*^Examples of high-risk patients include those with traumatic brain injury, severe traumatic burn injury, hypertrophic osteoarthritis, ankylosing spondylitis, diffuse idiopathic skeletal hyperostosis, or prior heterotopic ossification

## Surgical Interventions

**Fig. 2:**
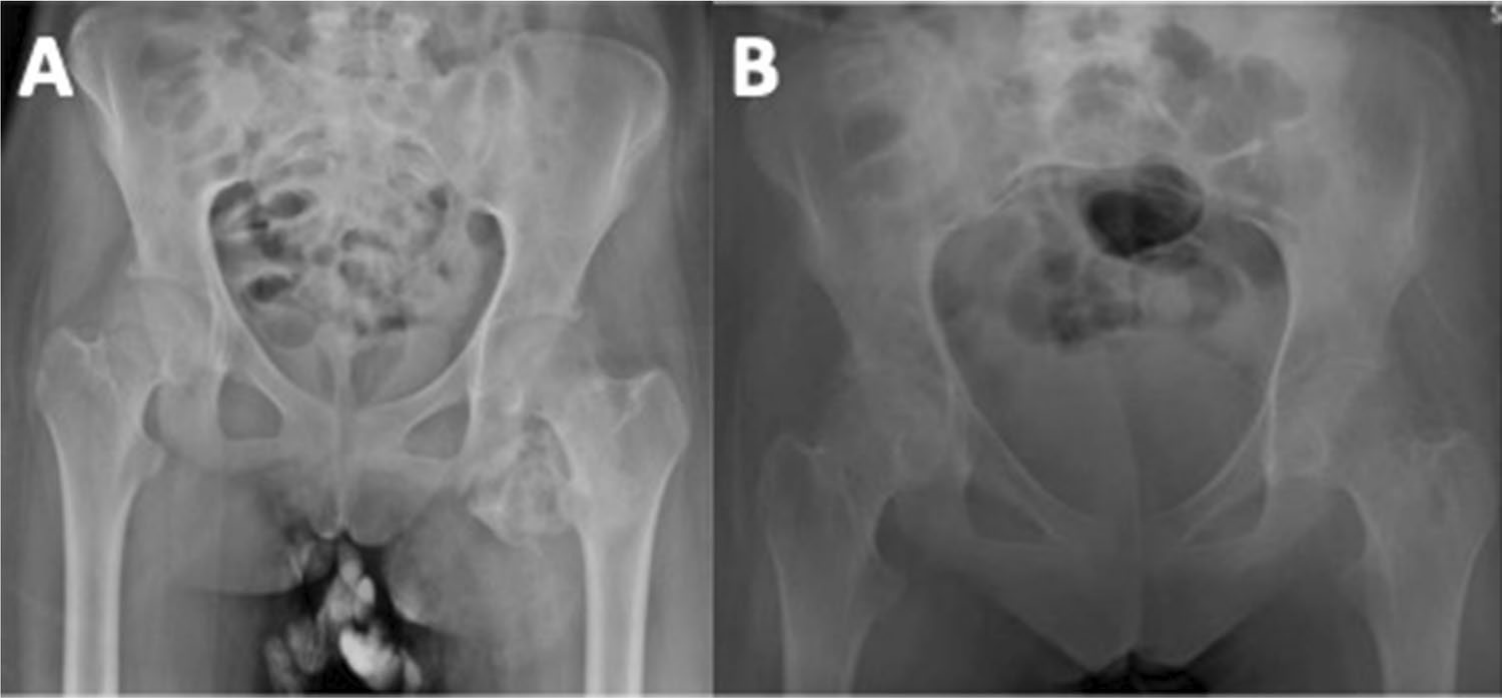
**A** AP pelvis radiographs demonstrating left hip heterotopic ossification in a 17-year-old female who 4 months prior had been involved in a motor vehicle accident suffering severe traumatic brain injury and **B** AP pelvis radiographs taken of the same patient in Fig. 2a after operative heterotopic ossification wide resection

Indications to proceed with surgical management are painful and restricted motion or overlying skin changes limiting function with many patients having tried non-operative modality to limit burden of symptoms. Ideal timing of surgical resection is debated though some waiting 1 year to allow osseous maturation and excision as surgical resection within 6 months has been associated with an increased risk of HO recurrence [60]. Advocates for earlier resection argue that it may prevent functional deficits seen from prolonged limited motion as HO matures [61]. Though uncommon, positron emission tomography (PET) CT with radiolabeled glucose (FDG) has been used in monitor HO flare-ups in the setting of genetic HO and can be considered a long with serum alkaline phosphatase to try and estimate HO maturity [62]. Goals of surgical management include complete resection which can involve extensile incision and wide exposures as incomplete resection of HO has been associated with recurrence [60]. After resection, early active range of motion is recommended to maintain range of motion after resection. As discussed above post-operative radiation or NSAIDs can be considered to prophylactically prevent recurrence of HO. Given the extensile approach and wide resection involved, excision of HO can carry a higher risk of blood loss and transfusion, infection, wound problems, and damage to surrounding neurovascular structures [4••, 63] (Fig. 2).

## Conclusion

Heterotopic ossification is a disabling condition that can occur in children. Risk factors for HO in pediatric patients include history of traumatic brain injury, trauma, and surgery. Diagnosis is made by physical exam and confirmed with imaging including radiographs and CT scan. Prophylaxis can be considered for high-risk surgical cases such as elbow or hip surgery, and it is typically comprised on indomethacin. However, the dosing and duration for prophylaxis in pediatric patients is unclear. We typically utilize 25 mg, three times a day for 5 days after higher risk procedures. Radiation is rarely considered in children but is an option in patients with significant risk. Surgical management is indicated for symptomatic HO, and typically occurs at least a year after onset to allow for maturation of the lesion and decrease risk of recurrence.
